# Tolerogenic XCR1^+^ dendritic cell population is dysregulated in HLA-B27 transgenic rat model of spondyloarthritis

**DOI:** 10.1186/s13075-019-1827-9

**Published:** 2019-02-04

**Authors:** Kétia Ermoza, Simon Glatigny, Nadège Jah, Vânia Camilo, Hendrick Mambu Mambueni, Luiza M. Araujo, Gilles Chiocchia, Maxime Breban

**Affiliations:** 1 INSERM U1173, UFR Simone Veil, Versailles-Saint-Quentin University, 2 avenue de la source de la Bièvre, 78190 Montigny le Bretonneux, France; 2 INFLAMEX, Laboratoire d’Excellence, Université Paris Diderot, Sorbonne Paris Cité, France; 3 Haematology-Immunology Division, Ambroise Paré Hospital (AP-HP), 9 avenue Charles de Gaulle, 92100 Boulogne-Billancourt, France; 4 Rheumatology Division, Ambroise Paré Hospital (AP-HP), 9 avenue Charles de Gaulle, 92100 Boulogne-Billancourt, France

**Keywords:** Spondyloarthritis, Ankylosing spondylitis, HLA-B27, Rat, Dendritic cell, XCR1

## Abstract

**Background:**

Spondyloarthritis (SpA) is a chronic inflammatory disease affecting primarily axial and peripheral joints and sometimes also extra-articular organs, such as the gut. Rats transgenic for HLA-B27 and human β2-microglobulin (B27-Tg rat) develop clinical manifestations resembling human disease. In this model, it has been shown that CD103^+^ conventional dendritic cells (cDCs) exhibited altered functions, likely promoting SpA development. CD4^−^ cDC subpopulation expressing XCR1, a chemokine receptor involved in their migration, have been described to be tolerogenic in steady state. Thus, in this study, we wished to examine the fate of XCR1^+^ cDCs in this animal model of SpA.

**Methods:**

cDC populations were isolated from the spleen, mesenteric lymph nodes (MLN), and colonic *lamina propria* from B27-TG and control nontransgenic (NTG) and/or HLA-B7 transgenic rats after collagenase digestion and density gradient and characterized with flow cytometry or real-time PCR. Migration of cDCs from intestinal mucosa to MLN was assessed, using TLR-7 stimulation with Resiquimod.

**Results:**

We observed a reduced frequency of cCD4^−^ DCs in B27-Tg rats, as compared to control rats. Furthermore, such decrease was not due to excessive death of CD4^−^ cDCs in B27-Tg rats. Interestingly, we observed a decrease frequency of the XCR1^+^ subpopulation among CD4^−^ cDCs in the spleen, MLN, and *lamina propria* from B27-Tg rats. Finally, after TLR-7 stimulation, the migration of XCR1^+^ cDCs to MLN was proportionally reduced in B27-Tg rats.

**Conclusion:**

Our results demonstrate for the first time a decreased proportion of the tolerogenic XCR1^+^ cDC subpopulation in SpA target organs in B27-Tg rat, which may affect the maintenance of self-tolerance and control of inflammation.

**Electronic supplementary material:**

The online version of this article (10.1186/s13075-019-1827-9) contains supplementary material, which is available to authorized users.

## Introduction

Spondyloarthritis (SpA) is a group of chronic inflammatory rheumatic diseases that primarily affect axial skeleton (i.e., spinal and sacro-iliac joints). Alongside axial disorder, peripheral articular manifestations often occur, including arthritis and enthesitis. Extra-articular features that are also frequently present include psoriasis, uveitis, and inflammatory bowel disease (IBD). It is thought that the development of SpA is determined by an interaction between particular genetic background and environmental factors [1].

Among predisposing factors, HLA-B27, a class I major histocompatibility complex (MHC) antigen, is the most strongly associated with SpA [1]. A HLA-B27/human β_2_-microglobulin (hβ2m) transgenic (B27-Tg) rat model was developed to investigate the implication of HLA-B27 in the pathogenesis of SpA. Strikingly, several lines of B27-Tg rat develop a spontaneous multisystem inflammatory disorder that recapitulates major SpA features, including arthritis, colitis, and psoriasiform skin and nail lesions [2].

Using this rat model, bone marrow cell transfer experiments showed that the expression of HLA-B27/hβ2m in myeloid cells was critical to trigger disease [3]. Furthermore, disease development required the presence of CD4^+^ T cells but expression of HLA-B27 by effector T cells was not required [4]. Selective expansion of Th17 was further shown, suggesting that this type of effector cell could be pathogenic in rat SpA [5, 6].

Among myeloid cells, dendritic cell (DC) is a variety of antigen-presenting cell that controls the priming and differentiation of naïve T cells and could therefore be involved in driving rat SpA [7]. Several subsets of rat DCs have been distinguished, according to their level of expression of αE_2_ integrin CD103, signal-regulatory protein α (SIRPα, CD172) and CD4. These include the plasmacytoid DCs (CD103^−^, SIRPα^+^, CD4^+^) and two subsets of conventional DCs (cDCs) expressing CD103: the SIRPα^−^, CD4^−^ cDC (CD4^−^ cDCs) and the SIRPα^+^, CD4^+^ cDC (CD4^+^ cDCs) corresponding to the cDC1and cDC2, respectively [8–10]. Both populations of cDCs exhibit distinct functions, the CD4^+^ cDCs being involved in T cell priming whereas the CD4^−^ cDCs is rather implicated in tolerance [8, 9, 11].

Previous studies have shown that splenic CD103^+^ cDCs from B27-Tg rat exhibited several aberrant functions, including impaired stimulation of T cells, alteration of cytoskeleton dynamics, reduced class II MHC expression, and enhanced apoptotic death [6, 12]. Interestingly, the CD103^+^CD172^low/−^ cDC population migrating through the intestinal mucosa-draining lymph appeared to be reduced in B27-Tg rat [6]. Such intestinal CD4^−^ cDCs, transporting absorbed fragments of apoptotic gut epithelial cells to the T cell area of MLN, have been implicated in the maintenance of self-tolerance [8]. Thus, considering its putative relevance for chronic inflammatory disease, it appeared of major interest to clarify which mechanism was behind such depletion. Theoretically, it could imply either increased cell death, as shown for other related B27-Tg rat DC populations, including Flt-3L-cultured bone marrow-derived DCs and splenic CD4^−^ cDCs, or impaired migration capacity [6, 12].

In the present study, we addressed such issues by analyzing CD103^+^ cDCs in the intestinal mucosa and lymphoid organs from B27-Tg rat. We confirmed a depletion of the CD4^−^ cDCs in the spleen of B27-Tg rat that was not related to increased cell death. Interestingly, the proportion of CD4^−^ cDCs expressing the X-C motif chemokine receptor 1 (XCR1), a chemokine receptor specifically expressed by a subpopulation of cDCs implicated in intestinal immune homeostasis [13, 14], was reduced in the spleen, the colonic *lamina propria*_*,*_ and the MLN from B27-Tg rat. The capacity of this subset to induce regulatory T cells (Treg) was impaired in B27-Tg rat. Moreover, the proportion of XCR1^+^ CD4^−^ cDC subpopulation, migrating from the *lamina propria* to MLN upon stimulation via TLR-7 was decreased in B27-Tg rat. Altogether, those results open novel avenues for deepening our understanding of the persistence of uncontrolled inflammatory response in B27-Tg rat model of SpA.

## Material and methods

### Animals

The HLA-B*2705/hβ_2_m disease-prone transgenic rats of the 33-3 line, bearing 55 copies of HLA-B*2705 and 28 copies of hβ_2_m, and the healthy HLA-B*0702/hβ_2_m transgenic rats of the 120-4 line bearing 52 copies of HLA-B*0702 and 26 copies of hβ_2_m, all on a Fisher (F344) background were bred and maintained under conventional conditions as previously described [5]. All the diseased B27-Tg rats had colitis and 20% of them had peripheral arthritis. Nontransgenic (NTG) littermates of the B27-Tg rats were used as controls. Age- and sex-matched rats were used in each experiment. Study procedures were approved by the Institutional Animal Experimentation Ethical Committee from the Faculty of Health Science Simone Veil.

### Isolation of lymphoid organs cells

To isolate cDCs, spleens, or mesenteric lymph nodes (MLN) were digested for 20 min at 37 °C with Collagenase D (2 mg/ml, Roche Diagnostics). Low-density cells were then collected after centrifugation on a 14.5% Nycodenz gradient (Nycomed). Splenic cells were then incubated with OX62 (anti-CD103 αE integrin) mAb-coated microbeads (Miltenyi Biotec) and positively selected with an Automacs Pro Separator to isolate DCs (Miltenyi Biotec). The level of purification checked by flow cytometry was routinely in the range of 70–90%. For MLN, low-density cells were analyzed by flow cytometry using appropriate markers (see below). After magnetic positive selection of CD103^+^ cDCs, CD4^−^ CD103^+^ cDC, or XCR1^+^ CD4^−^ CD103^+^ subsets were further isolated using the appropriate gating strategy with the BD Aria III cells sorter (BD), as required. Naïve CD4^+^ CD25^−^ CD62L^High^ T cells were isolated from MLN single cell suspension, using the appropriate gating strategy with the BD Aria III cells sorter (BD).

### Isolation of colonic *lamina propria* cells

Isolation of mononuclear cells from the proximal colon was performed following a procedure adapted from Weigmann et al. 2007 [15]. The proximal colon was identified by its morphology: it exhibits folds in an oblique direction corresponding to that of the arteries and the colonic wall in this colonic segment is thicker than below. The entire length of the proximal colon was harvested, by sectioning its proximal junction with the cæcum and its distal extremity. Proximal colon was open longitudinally and predigested with PBS containing EDTA (0.5 mM) for 20 min. It was then cut into small pieces and digested under agitation with Collagenase VIII (Roche) at 375 μg/ml, DNase I (Roche) at 60 U/ml and Dispase (Roche) at 10 mg/mL, in 10 ml of RPMI during 30 min at 37 °C. Finally, the released cells were loaded onto a Percoll (GE Healthcare) gradient and centrifuged. The cells between 40 and 80% Percoll were collected and used as *lamina propria* mononuclear cells.

### Flow cytometry

Cells were stained with fluorochrome-labeled monoclonal antibodies (mAbs): phycoerythrin (PE)/Cy7-conjugated or fluorescein isothiocyanate (FITC)-conjugated anti-rat CD4 (OX-35), PE-conjugated anti-rat CD45RC (OX-22), FITC-conjugated anti-rat TCRα/β (R73), and PE-conjugated anti-rat CD25 (OX-39) from Becton Dickson (BD) Bioscience; Brilliant Violet 510-conjugated anti-rat XCR1 (ZET) and allophycocyanin (APC)-conjugated anti-rat CD103 (OX-62) from BioLegend; eFluor 660-conjugated anti-rat CD62L (OX-85) from Thermo Fisher. When needed, surface markers were stained using appropriate mAbs cocktail prior to fixation and permeabilization with FoxP3/Transcription Factor Staining Buffer Kit according to the manufacturer instructions (eBioscience), using PE-eFluor 610-conjugated anti-FoxP3 (FJK-16 s) from Thermo Fisher. The cells were washed and suspended in PBS-FCS 2%. They were analyzed using a LSRIII Fortessa flow cytometer (BD). Data were analyzed using FlowJo software (TreeStar). A FACSAria instrument (BD) was used for flow cytometry sorting of the CD4^−^ and XCR1^+^ cDC subsets and the CD4^+^ CD62L^high^ TCRα/β^+^ T cells. For death/apoptosis detection, cells were stained with Violet 450 or APC-conjugated Annexin V in 100 μl of Annexin buffer (BD) and propidium iodide (PI) for 20 min at room temperature in the dark according to the manufacturer protocol (BD Biosciences).

### Quantitative real-time polymerase chain reaction (qRT-PCR)

For gene expression study, total RNA was isolated from purified CD4^−^ cDCs using a RNeasy kit (Qiagen), genomic DNA was eliminated by desoxyribonuclease treatment (RNase-Free DNase set; Qiagen), and samples were immediately stored at − 80 °C. The RNA quality was measured using a LabCHIP GX (PerkinElmer), and RNA concentration was measured with LabCHIP DS (PerkinElmer). Levels of messenger RNA (mRNA) of the tested genes were quantified by qRT-PCR (CFX384 Touch Real-Time PCR Detection System; BioRad) using iTaq™ Universal SYBR® Green Supermix (BioRad). Thermocycling included incubation at 95 °C for 10 min, followed by a two-step PCR program of 95 °C for 15 s and 55 °C for 60 s and extension at 72 °C for 10 s, for a total of 45 cycles. The total amount of mRNA was normalized across samples according to endogenous *glyceraldehyde-3-phosphate dehydrogenase* (*Gapdh*), *hypoxanthine phosphoribosyltransferase 1* (*Hprt1*), and *succinate dehydrogenase complex subunit A* (*Sdha*) mRNA. The forward (FW) and reverse (RV) primer sequences were as follows: for *Xcr1*, FW cgtgacatggactcagactc, and RV ccaccaggctgaggagaaat; for *Gapdh*, FW acgcaagcaaggatactgag and RV ggatggaaattgtgagggaga; for *Hprt1*, FW tttgtgtcatcagcgaaagtg and RV atggccacaggactagaac; for *Sdha*, FWgctgtgtcgctgatcgg and RV cagaagatccagtgcaaata. Relative gene expression was calculated using the ΔΔCt method, with *Hprt*1, *Sdha*, and *Gapdh* as housekeeping genes. For this, the mean Ct was calculated from *Hprt1*, *Sdha*, and *Gapdh*. The results were expressed as arbitrary unit (AU).

### Cell cultures

Cell cultures were performed in RPMI 1640 medium with GlutaMax I (Life Technologies) supplemented with 10% fetal calf serum, streptomycin (100 μg/ml), 2% sodium pyruvate, 0.05 mM 2-mercaptoethanol, and 5 mM HEPES (complete medium). Sorted splenic XCR1^+^ CD4^−^ cDCs were matured overnight in the presence of rat granulocyte-macrophage colony-stimulating factor (GM-CSF; 100 ng/ml), then co-cultured with sorted CD4^+^ CD25^−^ CD62L^high^ naïve T cells previously labeled with CellTrace Violet (5 μM, according to the CellTrace^TM^ Cell Proliferation Kit instructions) from Thermo Fisher) in the presence of anti-TCRα/β (R73) mAb for 6 days, in 96-well round-bottomed plates in 200 μl complete medium. The DC:T cell ratio was 1:5. After 6 days of culture, cells were stained with a live/dead marker, anti-CD4, and anti-FoxP3 Ab. Proliferation of T cells was assessed by FACS, by evaluating the dilution of CellTrace Violet in TCRα/β^+^ cells in live CD4+ T cells.

### Migration of DCs to MLN following Resiquimod feeding

Intestinal DC migration was assessed using the procedure described by Yrlid and al. 2006 [16].

Briefly, paired NTG and B27-Tg rats were fed with either 50 μg of Resiquimod (R-848, Sigma), a TLR-7 ligand, or PBS. Five hours later, the rats were sacrificed and their MLN harvested for cDC enumeration with flow cytometry, as described above. The ratio of cDC number collected in MLN after Resiquimod over PBS feeding was taken as an indication of the migratory stimulation effect of Resiquimod.

### Statistical analysis

Data were expressed as the mean ± SEM and analyzed using unpaired Student’s *t* test (two groups) or one-way ANOVA (three groups). *P* values less than 0.05 were considered significant. GraphPad Prism software was used for analyses.

## Results

### CD103^+^ CD4^−^ cDCs are depleted in B27-Tg rat spleen without increased cell mortality

**Fig. 1:**
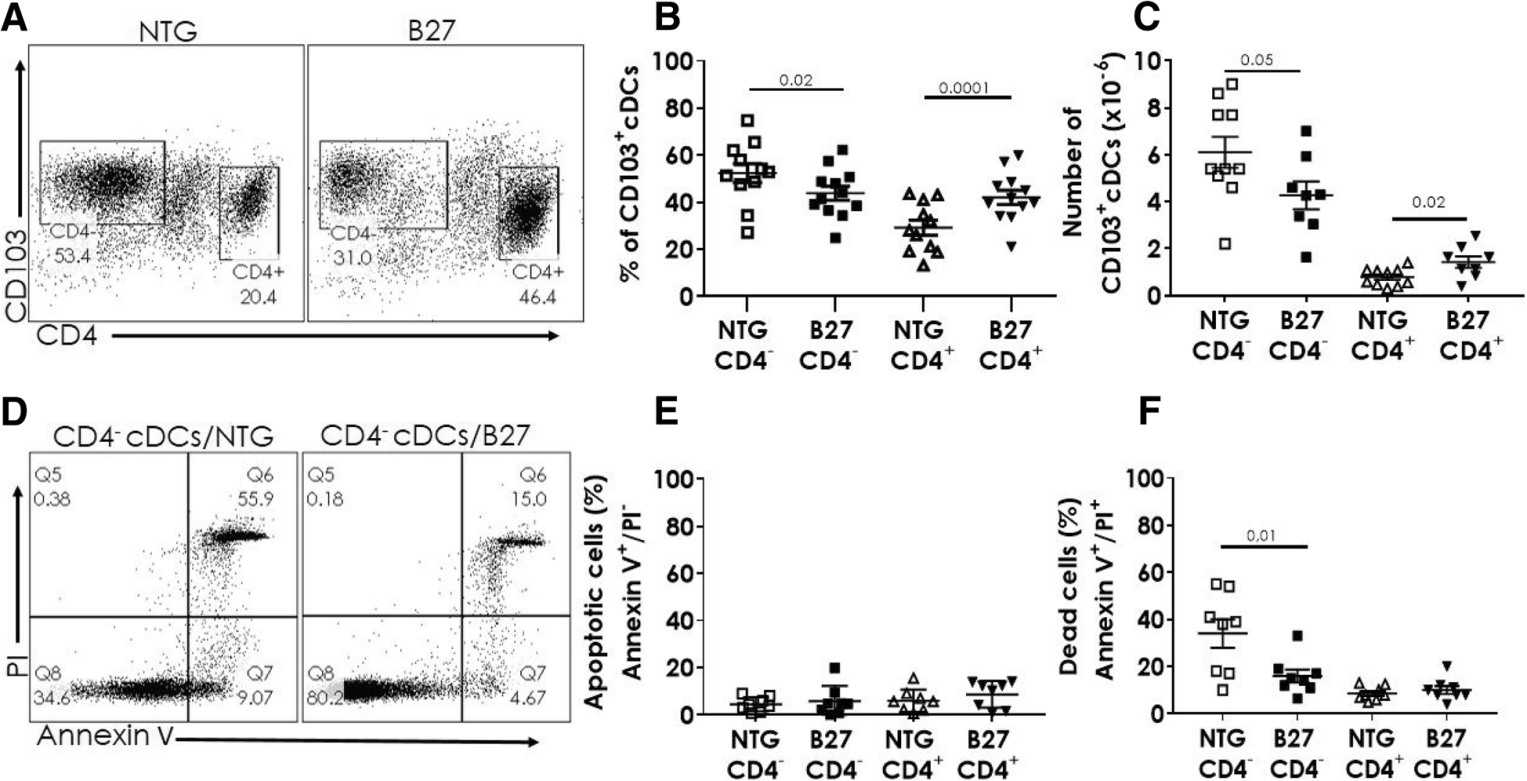
CD103^+^CD4^−^ cDCs are depleted in the spleen of B27-Tg rat. Freshly isolated CD103^+^ splenic cDCs from 6-month-old NTG and B27-Tg rats were analyzed by flow cytometry. **a** Representative dot-plots showing the expression of CD4 among CD103^+^ cDCs. **b** The graph shows the frequency of CD4^−^ and CD4^+^ cDCs among CD103^+^ cDCs in NTG and B27-Tg rats. **c** The graph shows the absolute numbers of CD4^−^ and CD4^+^ CD103^+^ cDCs in NTG and B27-Tg rats. **d** Representative dot-plots showing live (Annexin V^−^/PI^−^), apoptotic (Annexin V^+^/PI^−^) and dead (Annexin V^+^/PI^+^) cell proportions among CD4^−^ splenic CD103^+^ cDCs. **e**, **f** The graphs show the frequency of apoptotic (Annexin V^+^/PI^−^) (**e**) and dead (Annexin V^+^/PI^+^) (**f**) CD4^−^ and CD4^+^ cDCs in NTG and B27-Tg rats. All experiments were repeated several times, including 8 rats per group. Bars show the mean ± SEM. Data were analyzed by unpaired Student’s *t* test

To test the hypothesis that the cDC profile might be altered in B27-Tg rats, we examined the distribution of ex vivo-sorted CD103^+^ splenic cDCs by flow cytometry. At 6 months, we observed an imbalance between the frequency of CD4^−^ DCs among CD103^+^ cDCs that was decreased whereas the frequency of CD4^+^ was conversely increased in B27-Tg rats with established disease, as compared to NTG controls (Fig. 1a, b). Therefore, the ratio of CD4^−^/CD4^+^ cDCs was significantly lower in B27-Tg as compared to NTG rats (4 in B27-Tg vs. 9 in NTG rat, *P* = 0.001; Additional file 1: Figure S1A). Absolute numbers of CD4^−^ and CD4^+^ cDC subpopulations were also significantly different between B27-Tg and NTG rats, mirroring their frequency variations (Fig. 1c).

To determine if the depletion of CD4^−^ cDCs in B27-Tg rats was due to an increased cell death, we stained ex vivo-sorted CD103^+^ DCs with Annexin V and PI. Surprisingly however, CD4^−^ cDCs from B27-Tg rats appeared to contain higher live (Annexin V^−^/PI^−^) and lower dead (Annexin V^+^/PI^+^) cell fractions than their NTG counterparts (Fig. 1d, e). The frequency of apoptotic CD4^−^ cDCs was not different between B27-Tg and NTG control rats. Regarding the CD4^+^ cDC subpopulation, there was no difference of distribution of live/death/apoptotic cells between B27-Tg and NTG control rats (Fig. 1e).

### The frequency of XCR1-expressing CD4^−^ cDCs is decreased in the spleen of B27-Tg rat and their capacity to induce Treg differentiation is impaired

Previous study showed that the migratory CD103^+^CD172^low/−^ DCs were likewise depleted from the afferent lymph in B27-Tg rats [6]. Since the splenic CD4^−^ cDC subpopulation also express CD103^+^ but not CD172, they could be the counterpart of the foregoing subset of migrating DCs [8]. Thus, we speculated that decreased splenic CD4^−^ cDC population in B27-Tg rat could reflect a more general defective migration towards lymphoid organs. Interestingly, XCR1 is a chemokine receptor expressed only by CD4^−^ cDCs in the rat, whose ligand named lymphotactin or XCL1 is produced by activated T cells and by natural killer cells [13, 14].

**Fig. 2:**
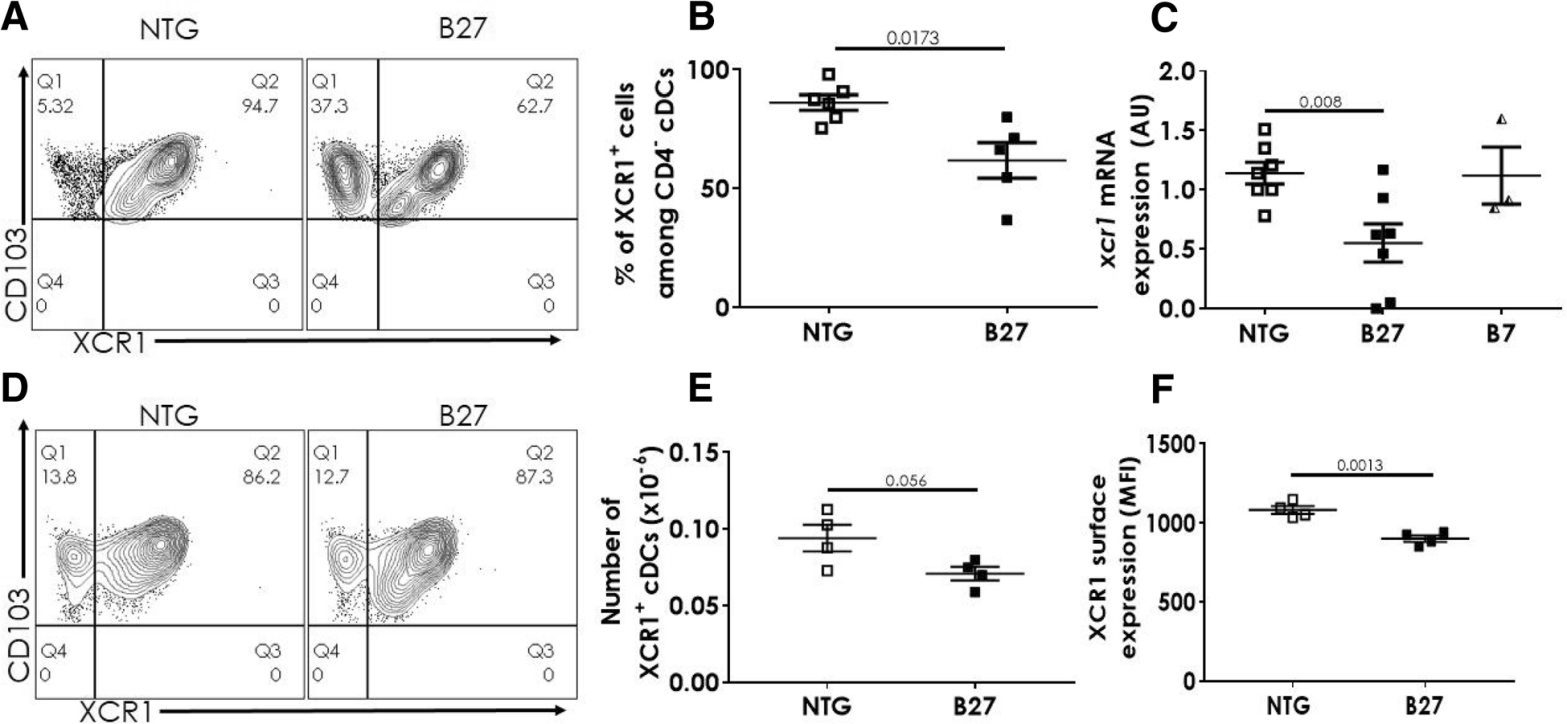
The frequency of XCR1-expressing CD4^−^ cDCs is decreased in the spleen of B27-Tg rat. **a**–**c** CD103^+^cDCs were isolated from the spleen of 6 month-old NTG, B27-Tg, and B7-Tg rats. XCR1 expression was evaluated in CD4^−^ cDCs by flow cytometry (**a**, **b**) or by qRT-PCR (**c**). **a** Representative dot-plots showing the expression of XCR1 on CD4^−^ CD103^+^ cDCs in NTG and B27-Tg rats. **b** The graph shows the frequency of XCR1^+^ among CD4^−^ CD103^+^ cDCs in NTG and B27-Tg rats. **c** The graph shows *Xcr1* mRNA levels in purified CD103^+^CD4^−^ splenic cDCs from NTG, B27-Tg, and B7-Tg rats, expressed in arbitrary units (AU). **d**–**f** CD103^+^cDCs were isolated from the spleen of 1-month-old NTG and B27-Tg rats. **d** Representative dot-plots showing the expression of XCR1 among CD4^−^ CD103^+^ cDCs in NTG and B27-Tg rats. **e** The graph shows the number of XCR1^+^ cDCs among CD4^−^ CD103^+^ cDCs in NTG and B27-Tg rats. **f** The graph shows the XCR1 mean fluorescence intensity (MFI) staining of XCR1^+^ CD4^−^ cDCs. Experiments were repeated 4–7 times. Bars show the mean ± SEM. Data were analyzed by unpaired Student’s *t* test (**b**, **e**, **f**) or one-way ANOVA (**c**)

We confirmed expression of XCR1 by flow cytometry on CD4^−^ splenic cDCs from F344 rats (Fig. 2a). In NTG rats, more than 80% of the CD4^−^ cDCs expressed XCR1 receptor (Fig. 2a, b). Similar result was found in B7-Tg rats (Additional file 2: Figure S2). In contrast, XCR1^+^ CD4^−^ cDC frequency was significantly lower, around 60%, in B27-Tg rats (Fig. 2a, b). Moreover, the expression of *Xcr1* mRNA determined by qRT-PCR on sorted splenic CD4^−^ cDCs was lower in B27-Tg rats, as compared to B7-Tg and NTG rats (Fig. 2c). The foregoing results were observed in 6-month-old rats with an already established disease. We next determined if similar differences could be observed prior to disease development. In 1-month-old premorbid rats, there was no difference in the frequency of XCR1^+^ CD4^−^ cDCs between B27-Tg and NTG rats, that was comparable to their frequency in the older NTG rats (Fig. 2d; Additional file 3: Figure S3A). However, there was a trend towards lower absolute numbers of CD4^−^ (*P* = 0.08; Additional file 3: Figure S3B) and particularly of XCR1^+^ (*P* = 0.056; Fig. 2e) cDCs in B27-Tg rat. Furthermore, the surface expression of XCR1 was reduced at 1 month in B27-Tg rat (Fig. 2f).

**Fig. 3:**
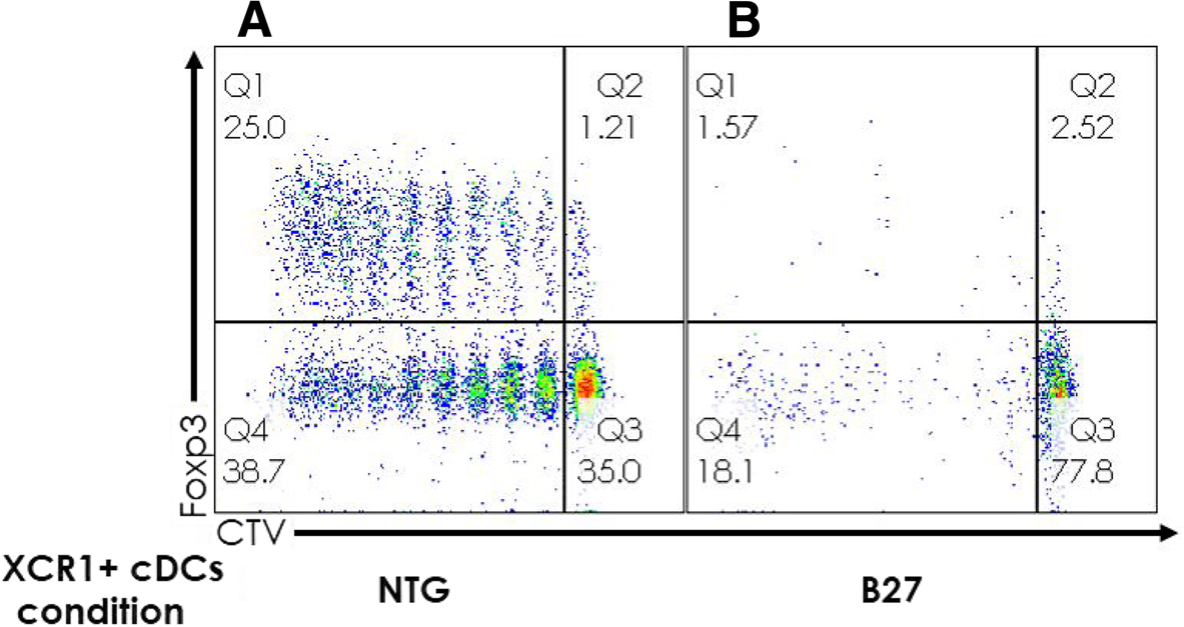
**a**, **b** Decreased capacity of splenic XCR1^+^ CD4^−^ cDCs from B27-Tg rats to support T cell proliferation and Treg differentiation. Sorted XCR1^+^ CD4^−^ CD103^+^ splenic cDCs from NTG (**a**) and B27-Tg (**b**) rats were cultured overnight with GM-CSF and tested the next day for their capacity to prime in vitro sorted naïve CD4^+^ CD25^−^ CD62L^high^ T cells isolated from NTG rat MLN (labeled with CellTrace Violet (CTV) to evaluate T cell proliferation) in the presence of anti-TCRα/β antibody. After 6 days of culture, cells were stained with a live/dead marker, anti-CD4, and anti-FoxP3 antibodies. The plots are gated on live CD4^+^ T cells and show the proliferation of CD4^+^ T cells (as dilution of CellTrace Violet) and FoxP3 expression. Results shown are representative of 2 independent experiments

Sorted splenic XCR1^+^ CD4^−^ cDCs from NTG rat co-cultured with naïve CD4^+^ T cells from NTG rat in the presence of anti-TCRα/β antibody supported T cell stimulation and Treg differentiation (Fig. 3a), whereas both functions were impaired with XCR1^+^ CD4^−^ cDCs from B27-Tg (Fig. 3b).

### The frequency of XCR1^+^ CD4^−^ cDCs is decreased in MLN from B27-Tg rat

**Fig. 4:**
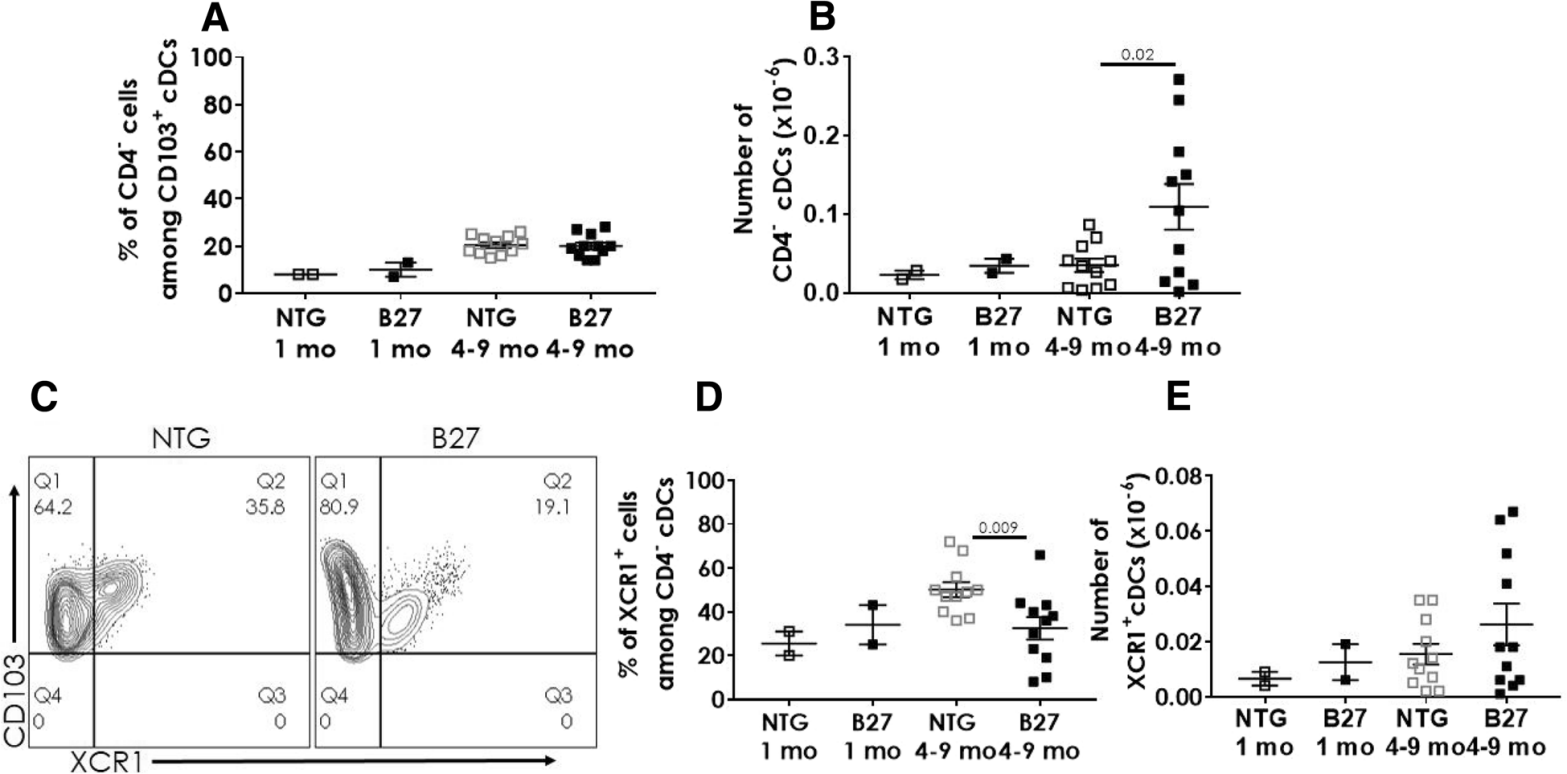
The proportion of XCR1^+^CD4^−^ cDCs is decreased in MLN from B27-Tg rats with established disease. Mononuclear cells were isolated from the MLN of NTG and B27-Tg rats at different ages (premorbid: 1 month, established disease: 4–9 months) and analyzed by flow cytometry. **a** The graph shows the frequency of CD4^−^ among CD103^+^ cDCs in MLN from NTG and B27-Tg at different ages. **b** The graph shows the absolute number of CD4^−^ cDCs in MLN from NTG and B27-Tg rats at different ages. **c**–**e** XCR1 expression was evaluated among CD4^−^ cDCs by flow cytometry. **c** Representative dot-plots showing the expression of XCR1 among CD4^−^ CD103^+^ cDCs in 8-month-old NTG and B27-Tg rats. **d** The graph shows the frequency of XCR1^+^ among CD4^−^ CD103^+^ cDCs in MLN from NTG and B27-Tg rats (**e**). The graph shows the absolute number of XCR1^+^ cDCs in MLN from NTG and B27-Tg rats at different ages. Bars show the mean ± SEM. Data were analyzed by unpaired Student’s *t* test

The depletion of XCR1^+^ DCs from the spleen of B27-Tg rats with established disease might be a consequence of their trapping in inflammatory tissues, such as the intestinal mucosa and/or draining MLN. We next examined the distribution of cDC populations in the MLN of B27-Tg rats of different ages (Fig. 4). The frequency and numbers of CD4^−^ (Fig. 4a, b) and XCR1^+^ (Fig. 4d, e) cDCs were similar between premorbid B27-Tg and NTG rats at 1 month. In B27-Tg rats with established disease, absolute numbers of CD4^−^ cDCs (*P* = 0.02; Fig. 4b), but not of XCR1^+^ cDCs (Fig. 4e) were significantly increased in B27-Tg rat. Therefore, the proportion of XCR1^+^ cDCs appeared significantly decreased in the B27-Tg rats with established disease (mean ± SEM: 32 ± 5.6%), as compared to NTG rats (50 ± 3.8%) (*P* = 0.009; Fig. 4c, d).

### Frequency of XCR1^+^ CD4^−^ cDC population is decreased in colonic *lamina propria* in B27-Tg rat

As expected, the mononuclear cell population isolated from the proximal colon-*lamina propria* was significantly increased in B27-Tg rats with established IBD (data not shown). Likewise, TCRα/β^+^ T cells were enriched in the B27-Tg rats (data not shown). The frequency and absolute numbers of CD103^+^ cDC population were also higher in B27-Tg rats (Additional file 4: Figure S4A and B).

**Fig. 5:**
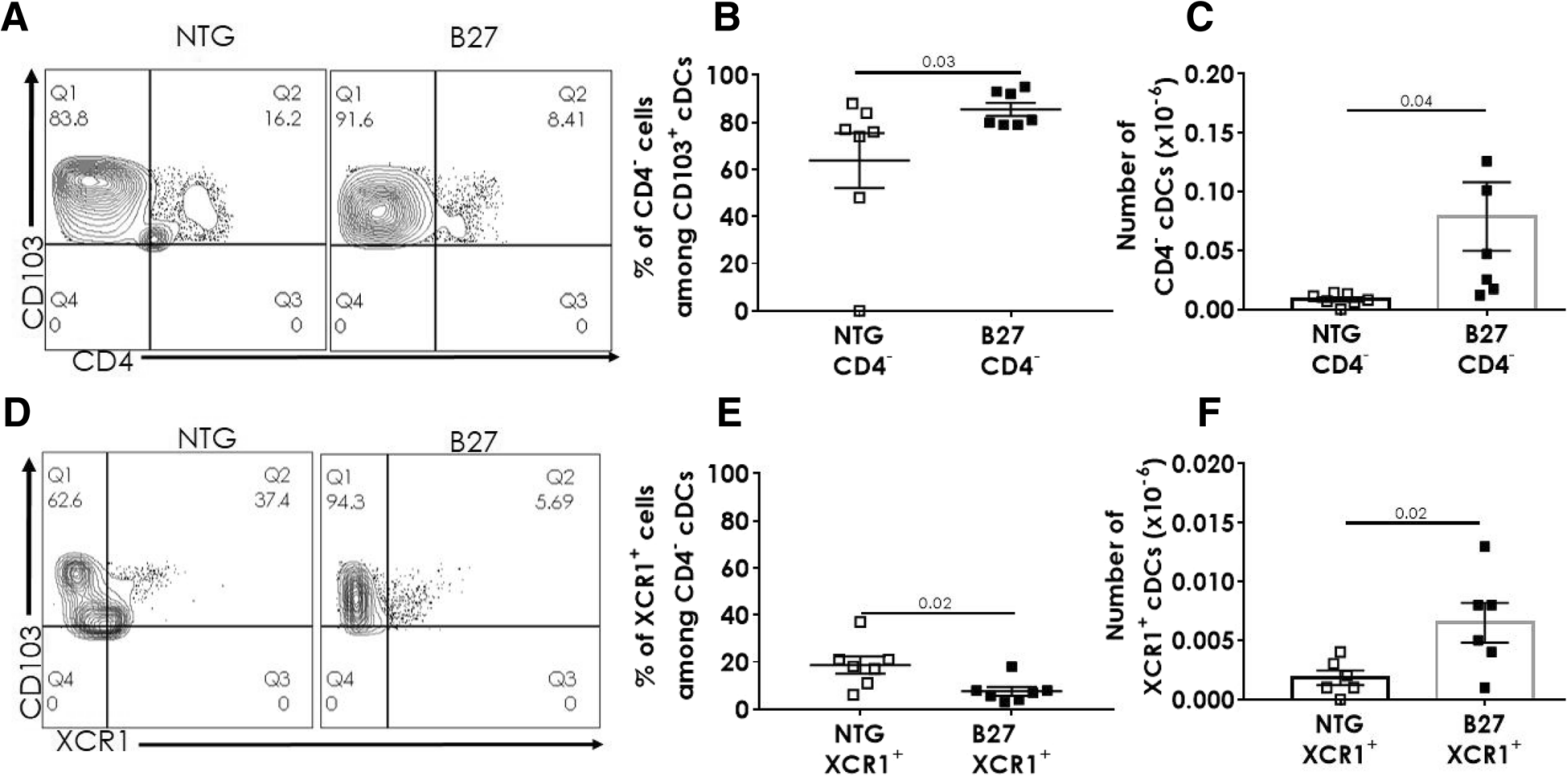
The XCR1^+^CD4^−^ cDC population is decreased in B27-Tg rat colonic *lamina propria*. Cells were isolated from the colonic *lamina propria* of 6–9-month-old rats, marked for CD103, CD4, and XCR1 and analyzed by flow cytometry. **a** Representative dot-plots showing the expression of CD4 among CD103^+^ cDCs in NTG and B27-Tg rats. **b**, **c** The graphs show the frequency (**b**) and absolute number (**c**) of CD4^−^ among CD103^+^ cDCs in NTG and B27-Tg rats. **d** Representative dot-plots showing the expression of XCR1 among CD103^+^ cDCs in NTG and B27-Tg rats. **e**, **f** The graphs show the frequency (**e**) and absolute number (**f**) of XCR1^+^ cDCs among CD103^+^ CD4^−^ cDCs in NTG and B27-Tg rats. The experiment was repeated 7 times. Bars show the mean ± SEM. Data were analyzed by unpaired Student’s *t* test

As in the spleen and MLN, CD103^+^ cDCs can be split into two distinct populations in the *lamina propria*, according to CD4 expression. Neither frequency nor numbers of CD4^+^ cDCs were different between B27-Tg and NTG rats in the colonic *lamina propria* (Additional file 4: Figure S4C and D). In contrast, both CD4^−^ DCs frequency and absolute numbers were significantly higher in B27-Tg rats with established disease than in NTG rats (Fig. 5b, c). Regarding the XCR1^+^ DCs population, it was numerically higher in B27-Tg than NTG rats (Fig. 5f), albeit its frequency was significantly lower among the CD4^−^ cDCs in the B27-Tg (7.6 ± 1.9%) than in the NTG rats (18.6 ± 3.5%) (*P* = 0.02; Fig. 5e).

### The proportion of XCR1^+^ cDCs migrating in response to TLR-7 stimulation is decreased in the B27-Tg rat

**Fig. 6:**
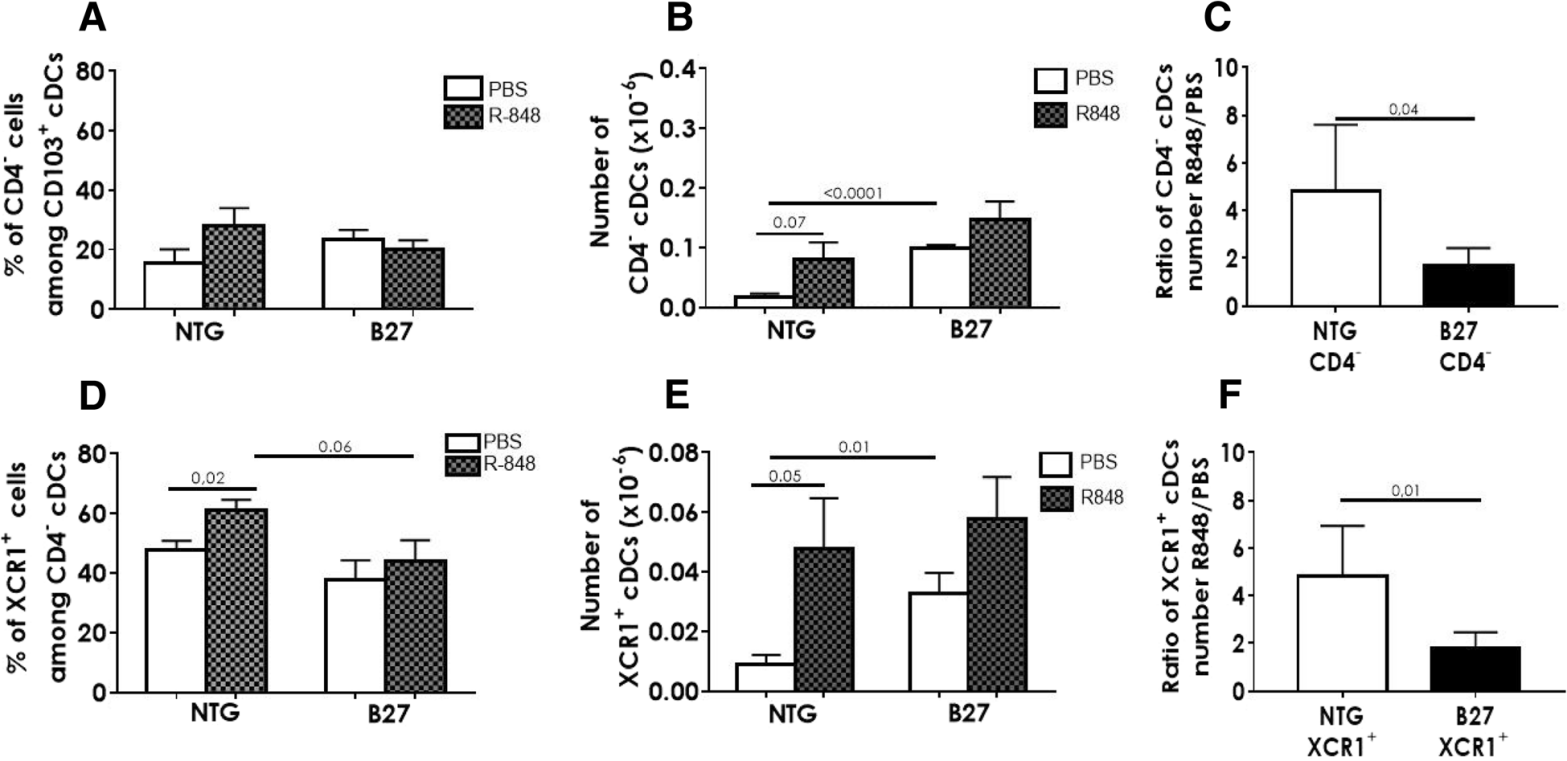
Intestinal XCR1^+^CD4^−^ cDCs in B27-Tg rat migrate less in response to TLR-7 stimulation. Low-density cells were isolated from MLN of 6 month-old NTG and B27-Tg rats that had been fed 5 h before with the TLR-7 agonist R-848 to activate DC migration from the intestine to the afferent lymph. Control NTG and B27-Tg rats received PBS. cDCs from MLN were analyzed by flow cytometry. **a**, **b** The graphs show the frequency (**a**) and absolute number (**b**) of CD4^−^ cDCs among CD103^+^ cDCs in R-848- and PBS-fed NTG and B27-Tg rats. **c** The graph shows the ratio of CD4^−^ cDCs number between R848- and PBS-fed conditions in NTG and B27-Tg rats. **d**, **e** The graphs show the frequency (**d**) and absolute number (**e**) of XCR1^+^ DCs among CD4^−^ CD103^+^ cDCs in R-848- and PBS-fed NTG and B27-Tg rats. **f** The graph shows the ratio of XCR1^+^ cDCs number between R848- and PBS-fed conditions in NTG and B27-Tg rats. This experiment was repeated 5 times. Bars show the mean ± SEM. Data were analyzed by unpaired Student’s *t* test

To test the migratory capacity of *lamina propria*-XCR1^+^ DCs, we fed 6-month-old rats with Resiquimod (R-848; 50 μg/ml), a TLR-7 ligand, or PBS. TLR-7 is known to be expressed on CD4^−^ cDCs and R-848 to induce the migration of intestinal DCs through afferent lymph to MLNs [16]. After 5 h, MLN low-density cells were isolated and cDC subpopulations frequency was evaluated. The frequency of MLN CD4^−^ cDCs among CD103^+^ cDCs was higher after R-848 (28%) than PBS (15.3%) feeding, in the NTG rats, albeit the difference was not statistically significant (Fig. 6a). Such difference was not apparent in the B27-Tg rat (Fig. 6a). Similarly, the numbers of CD4^−^ cDCs in MLN appeared higher after R-848 feeding in NTG (0.8 ± 0.3 × 10^5^ cells), as compared to PBS conditions (0.2 ± 0.06 × 10^5^ cells), with borderline statistically significant difference (*P* = 0.07; Fig. 6b). No significant difference was observed in B27-Tg rats after R-848 feeding (1.5 ± 0.3 × 10^5^ cells), as compared to PBS condition (1 ± 0.06 × 10^5^ cells). Hence, the ratio of CD4^−^ DCs collected after R-848/PBS was significantly lower in B27-Tg (1.7 ± 0.3) than in NTG (4.8 ± 1.3) rats (*P* = 0.04; Fig. 6c). Considering that the CD4^−^ cDC population was enriched in B27-Tg rat lamina propria (Fig. 5c), this result indicates that the proportion of CD4^−^ cDCs migrating to the MLN after R-848 feeding was lower in B27-Tg than NTG rats.

We next examined XCR1^+^ DCs frequency among CD4^−^ cDCs. It was again significantly greater in R-848-fed than in PBS-fed NTG rats (Fig. 6d). In contrast, XCR1^+^ cDC frequency appeared lower in B27-Tg than in NTG rats fed with R-848, with borderline statistical significance (*P* = 0.06) and was not different from PBS-fed B27-Tg rats (Fig. 6d). The numbers of XCR1^+^ cDCs in MLN were higher after R-848 feeding in NTG (5 ± 2 × 10^4^ cells) (*P* = 0.05, Fig. 6e), as compared to PBS conditions (0.9 ± 0.3 × 10^4^ cells). There was no significant difference in B27-Tg rats, after R-848 feeding (6 ± 1.4 × 10^4^ cells) as compared to PBS conditions (3 ± 0.7 × 10^4^ cells) (Fig. 5e). Therefore, the ratio of XCR1^+^ cDCs collected after R-848/PBS was again significantly lower in B27-Tg than in NTG rats (*P* = 0.01; Fig. 6f), indicating that a smaller proportion of intestinal mucosal XCR1^+^ cDCs reached the MLN after R-848, in B27-Tg rat than in NTG rat.

Interestingly, the migration of XCR1^−^ CD4^−^ cDCs was not significantly different between B27-Tg and NTG rats (Additional file 5: Figure S5A and B).

## Discussion

At the cross-talk between innate and adaptive immunity, DCs play a central regulatory role in immune response as well as tolerance [11]. This, along with the observation that HLA-B27/hβ2m-expressing myeloid-derived cell was critical to trigger inflammatory disorder in B27-Tg rat, justified to explore the potential implication of cDCs in the triggering of this SpA model. Indeed, several aberrant functions of the B27-Tg rat CD103^+^ cDCs were previously reported in vitro, including decreased motility, increased susceptibility to cell death, and induction of a pro-inflammatory Th17 bias [5, 6, 12]. Moreover, the CD103^+^ CD4^−^ cDCs were reported as depleted in the afferent lymph of B27-Tg rats [6]. Given that this population of cDCs is thought to exert tolerogenic effect, it was important to further clarify its fate in the B27-Tg rat model [8, 17].

Consistent with the foregoing hypothesis, we first observed that CD4^−^ cDCs were also depleted in B27-Tg rat spleen. However, contrary to our expectations, given their previously identified increased susceptibility to death in vitro [12], such depletion could not be attributed to higher mortality of the freshly isolated CD4^−^ cDCs. We then hypothesized that a selective homing defect of the CD4^−^ cDC population could rather explain their depletion from the spleen. Thus, we were interested in XCR1, a chemokine receptor exclusively expressed by most of the splenic CD4^−^ cDCs and known to provoke the migration of this DC subset in response to its ligand, XCL1 [18].

Indeed, nearly all the CD4^−^ cDCs in the spleen from control NTG F344 rats expressed XCR1. However, in B27-Tg rat with established disease, as many as 40–50% of the CD4^−^ cDCs did not express XCR1 in the spleen. Even at 1 month, i.e., before any overt disease manifestation, there was a trend towards fewer numbers of XCR1^+^ cDCs expressing lower levels of XCR1 in the spleen of B27-Tg rats, suggesting that depletion of XCR1^+^ cDCs is an early event in the B27-Tg rat disease process. In addition, the splenic XCR1^+^ cDCs from B27-Tg rats were impaired in their capacity to support the proliferation of naïve CD4^+^ T cells and their differentiation into Treg.

We next studied CD4^−^ cDCs in the colonic mucosa, the earliest site of inflammation, and in the draining MLN where Th17 cells are known to be expanded in B27-TG rat [5, 19]. Contrary to the spleen, the numbers of CD4^−^ cDCs were increased both in the colonic *lamina propria* and in the MLN in B27-TG rat with established disease. This could indicate that CD4^−^ cDCs localized to the inflammatory site, rather than to the spleen. Interestingly, however, the proportion of XCR1^+^ cDCs was again reduced in both sites.

Finally, to test whether XCR1^+^ CD4^−^ cDCs exhibited a homing defect in B27-Tg rat, we used R-848, a TLR-7 ligand known to induce the migration of DCs from intestinal *lamina propria* to MLN. Indeed, we observed an increased number of XCR1^+^ CD4^−^ cDCs in MLN from NTG rat, 5 h after R-848 feeding. However, such increase was not significant in the B27-Tg rat and less important than in control NTG rat, even though the number of XCR1^+^ CD4^−^ cDCs was increased in B27-Tg rat *lamina propria*. Such decreased migration of XCR1^+^ CD4^−^ cDCs appeared consistent with the depletion of CD4^−^ cDCs previously reported in the afferent lymph of lymphadenectomized B27-Tg rat [6]. It has been shown that splenic cDCs from B27-Tg rats had impaired motility due cytoskeleton alterations [12]. This could account for the defective migration observed in this study.

The role of XCR1^+^ cDCs in controlling inflammation has been previously highlighted [14]. One of the mechanisms by which XCR1^+^ DCs could contribute to control excessive inflammation in the gut mucosa is the production of transforming growth factor-β1 and retinoic acid from vitamin A, thereby promoting the induction of peripheral Treg [20]. Indeed, in murine experimental model of colitis, selective deletion of this subset of DCs was associated with an expansion of XCR1^−^ CD103^+^ cDC2 in the gut mucosa, decreased migration of those cells to MLN, increased colitis severity, and preferential polarization of effector T cells towards Th17 cells [14]. Hence, the reduced proportion of XCR1^+^ DCs observed in the B27-Tg rat and their impaired capacity to support Treg differentiation could likewise contribute to the abnormal expansion of Th17 cells and chronic inflammation [5]. Moreover, XCR1^+^ cDCs are critical for keeping intestinal intraepithelial lymphocyte (IEL) population integrity that is important to prevent intestinal inflammation [14, 21, 22]. Indeed, selective depletion of XCR1^+^ cDCs in the foregoing mouse model induced a significant impairment of CD8αα^+^ IEL [14]. Interestingly, a recent study reported a decrease of colonic intraepithelial lymphocyte especially the CD8αα^+^ IEL, in SpA patients’ colonic mucosa [23]. This diminished CD8αα^+^ IEL could likewise reflect an impaired XCR1^+^ cDCs in SpA patient.

Nevertheless, the mechanism accounting for decreased proportion of XCR1^+^ cDCs in B27-Tg rat remains unknown. Noteworthy, XCR1 expression in cDCs is closely correlated with their ability to cross-present antigen, i.e., to process extra-cellularly acquired antigens through class I MHC presentation pathway, a function that has not yet been explored in B27-Tg rat [13]. By doing so, XCR1^+^ cDCs engage in mutual interactions with CD8^+^ T cells that upon activation produce XCL1, which in turn fosters the communication of CD8^+^ T cells with XCR1^+^ cDCs. Moreover, the capacity of XCR1^+^ cDCs to take up certain forms of antigen such as *Listeria monocytogenes* and to interact with XCL1-producing natural killer (NK) cells and CD8^+^ T cells might be critical for defense against this type of pathogen [13, 24]. Interestingly, B27-Tg rat were shown to be exquisitely sensitive to infection by *L. monocytogenes* which might reveal deficient XCL1-XCR1 system of defense against certain type of intra-cellular microbes [25].

## Conclusion

Our results show a significant decrease of the tolerogenic XCR1^+^ DC subpopulation in B27-Tg rat lymph nodes and spleen which may impact the maintenance of self-tolerance and the control of inflammation.

## Additional files

Additional file 1: **Figure S1.** Ratio of CD4-/CD4+ cDCs is reduced in the spleen but not in MLN from B27-Tg rat. Splenic CD103^+^ cDCs were isolated from NTG and B27-Tg rats with established disease and analyzed by flow cytometry. The ratio of CD103^+^CD4^−^/CD103^+^CD4^+^ cDCs was analyzed among live cells in the spleen (A) or in MLN (B). This experiment was repeated 10 times. Bars show the mean ± SEM. Data were analyzed by unpaired Student’s *t* test. (PDF 39 kb)

Additional file 2: **Figure S2.** Frequency of splenic XCR1^+^ cDCs is similar in B7-Tg rats than in NTG rats at 6 month. CD103^+^cDCs were isolated from the spleen of 6-month-old NTG, B7-Tg, and B27-Tg rats. XCR1 frequency was evaluated in CD4^−^ cDCs by flow cytometry. The graph shows the frequency of XCR1^+^ cDCs among CD4^−^ CD103^+^ cDCs in NTG, B7-Tg, and B27-Tg rats. This experiment was repeated 3 times. Bars show the mean ± SEM. (PDF 31 kb)

Additional file 3: **Figure S3.** The number of splenic XCR1^+^ cDCs is marginally decreased in B27-Tg rat at 1 month. CD103^+^ cDCs were isolated from the spleen of 1-month-old NTG and B27-Tg rats. (A) The graph shows the frequency of XCR1^+^ cDCs among CD4^−^ CD103^+^ cDCs in NTG and B27-Tg rats. (B) The graph shows the number of CD4^−^ cDCs in NTG and B27-Tg rats. This experiment was repeated 4 times. Bars show the mean ± SEM. Data were analyzed by unpaired Student’s *t* test. (PDF 40 kb)

Additional file 4: **Figure S4.** The whole population of CD103^+^ cDCs is increased but not the CD4^+^ cDCs, in B27-Tg rat colonic *lamina propria*. Mononuclear cells were isolated form the colonic *lamina propria* and stained with anti-CD103 and anti-CD45 mAbs. The graphs show the frequency (A) and absolute number (B) of CD103^+^ cDCs among CD45RC^−^ cDCs in NTG and B27-Tg rats. The graphs show the frequency (C) and absolute number (D) of CD4^+^ cDCs among CD103 ^+^ cDCs in NTG and B27-Tg rats. This experiment was repeated 7–8 times. Bars show the mean ± SEM. Data were analyzed by unpaired Student’s *t* test. (PDF 66 kb)

Additional file 5: **Figure S5.** Number of XCR1^−^ cDCs in 6-month-old B27-Tg rat MLN after TLR-7 stimulation. Low-density cells were isolated from 6-month-old NTG and B27-Tg rats MLN that had been fed 5 h before with the TLR-7 agonist R-848 to activate DC migration from the intestine to the afferent lymph. Control NTG and B27-Tg rats received PBS (A) The graph shows the absolute number of XCR1^−^ cDCs among CD4^−^ CD103^+^ cDCs in R-848- and PBS-fed NTG and B27-Tg rats. (B) The graph shows the ratio of XCR1^−^ cDC numbers between R848- and PBS-fed conditions in NTG and B27-Tg rats. This experiment was repeated 5 times. Bars show the mean ± SEM. Data were analyzed by unpaired Student’s *t* test. (PDF 42 kb)

## Abbreviations

B27-Tg: HLA-B27 and human β2-microglobulin transgenic
cDCs: Conventional dendritic cells
Ct: Cycle threshold
CTV: CellTrace Violet
EDTA: Ethylenediaminetetraacetic acid
FCS: Fetal calf serum
Fig.: Figure
FITC: Fluorescein isothiocyanate
Flt-3L: FMS like tyrosine 3 receptor ligand
GAPDH: Glyceraldehyde-3-phosphate dehydrogenase
GM-CSF: Granulocyte-macrophage colony-stimulating factor
HPRT: Hypoxanthine phosphoribosyltransferase 1
hβ2m: Human β2-microglobulin
IBD: Inflammatory bowel disease
IEL: Intraepithelial lymphocyte
*L. monocytogenes*: *Listeria monocytogenes*
mAb: Monoclonal antibody
MHC: Major histocompatibility complex
MLN: Mesenteric lymph node
mRNA: Messenger ribonucleic acid
NK: Natural killer
NTG: Nontransgenic
PBS: Phosphate buffered saline
PE: Phycoerythrin
PI: Propidium iodide
qRT-PCR: Quantitative real-time polymerase chain reaction
R-848: Resiquimod
SDHA: Succinate dehydrogenase complex subunit A
SEM: Standard error of mean
SIRPα: Signal-regulatory protein alpha
SpA: Spondyloarthritis
Th17: T helper cell 17
TLR-7: Toll-like receptor 7
Treg: Regulatory T cells
XCL1: X-C motif chemokine ligand 1
XCR1: X-C motif chemokine receptor 1

## References

[1] Taurog JD, Chhabra A, Colbert RA (2016). Ankylosing spondylitis and axial spondyloarthritis. N Engl J Med.

[2] Hammer RE (1990). Spontaneous inflammatory disease in transgenic rats expressing HLA-B27 and human B2m: an animal model of HLA-B27-associated human disorders. Cell.

[3] Breban M, Hammer RE, Richardson JA, Taurog JD (1993). Transfer of the inflammatory disease of HLA-B27 transgenic rats by bone marrow engraftment. J Exp Med.

[4] Breban M, Fernandez-Sueiro JL, Richardson JA, Hadavand RR. T cells in inflammatory disease of B27 transgenic rats. J Immunol. 1996:156–803. 8543835

[5] Glatigny S (2012). Proinflammatory Th17 cells are expanded and induced by dendritic cells in spondylarthritis-prone HLA-B27-transgenic rats. Arthritis Rheum.

[6] Utriainen L (2012). Expression of HLA-B27 causes loss of migratory dendritic cells in a rat model of spondylarthritis. Arthritis Rheum.

[7] Melis L, Elewaut D (2009). Progress in spondylarthritis. Immunopathogenesis of spondyloarthritis: which cells drive disease?. Arthritis Res Ther.

[8] Huang F-P (2000). A discrete subpopulation of dendritic cells transports apoptotic intestinal epithelial cells to T cell areas of mesenteric lymph nodes. J Exp Med.

[9] Voisine C, Hubert F-X, Trinite B, Heslan M, Josien R (2002). Two phenotypically distinct subsets of spleen dendritic cells in rats exhibit different cytokine production and T cell stimulatory activity. J Immunol.

[10] Guilliams M (2014). Dendritic cells, monocytes and macrophages: a unified nomenclature based on ontogeny. Nat Rev Immunol.

[11] Iberg CA, Jones A, Hawiger D (2017). Dendritic cells as inducers of peripheral tolerance. Trends Immunol.

[12] Dhaenens M (2009). Dendritic cells from spondylarthritis-prone HLA-B27-transgenic rats display altered cytoskeletal dynamics, class II major histocompatibility complex expression, and viability. Arthritis Rheum.

[13] Kroczek RA, Henn V. The role of XCR1 and its ligand XCL1 in antigen cross-presentation by murine and human dendritic cells. Front Immunol. 2012;3:14 10.3389/fimmu.2012.00014 PMC3342032 22566900

[14] Ohta T (2016). Crucial roles of XCR1-expressing dendritic cells and the XCR1-XCL1 chemokine axis in intestinal immune homeostasis. Sci Rep.

[15] Weigmann B (2007). Isolation and subsequent analysis of murine lamina propria mononuclear cells from colonic tissue. Nat Protoc.

[16] Yrlid U (2006). Regulation of intestinal dendritic cell migration and activation by plasmacytoid dendritic cells, TNF- and type 1 IFNs after feeding a TLR7/8 ligand. J Immunol.

[17] Milling SWF (2009). Steady-state migrating intestinal dendritic cells induce potent inflammatory responses in naive CD4+T cells. Mucosal Immunol.

[18] Geyer H (2014). Cytomegalovirus expresses the chemokine homologue vXCL1 capable of attracting XCR1+ CD4− dendritic cells. J Virol.

[19] DeLay ML (2009). HLA-B27 misfolding and the unfolded protein response augment interleukin-23 production and are associated with Th17 activation in transgenic rats. Arthritis Rheum.

[20] Denning TL (2011). Functional specializations of intestinal dendritic cell and macrophage subsets that control Th17 and regulatory T cell responses are dependent on the T cell/APC ratio, source of mouse strain, and regional localization. J Immunol.

[21] Poussier P, Ning T, Banerjee D, Julius MA (2002). Unique subset of self-specific intraintestinal T cells maintains gut integrity. J Exp Med.

[22] Das G (2003). An important regulatory role for CD4+CD8 T cells in the intestinal epithelial layer in the prevention of inflammatory bowel disease. Proc Natl Acad Sci.

[23] Regner EH, et al. Functional intraepithelial lymphocyte changes in inflammatory bowel disease and spondyloarthritis have disease specific correlations with intestinal microbiota. Arthritis Res. Ther. 2018;20:149. 10.1186/s13075-018-1639-3 PMC6053728 30029674

[24] Crozat K (2010). The XC chemokine receptor 1 is a conserved selective marker of mammalian cells homologous to mouse CD8α ^+^ dendritic cells. J Exp Med.

[25] Warner TF, Madsen J, Starling J, Taurog JD, Balishtt E (1996). Human HLA-B27 gene enhances susceptibility of rats to oral infection by listeria monocytogenes. AmJPathol.

