# Supplementary figures and images for: Tolerogenic XCR1+ dendritic cell population is dysregulated in HLA-B27 transgenic rat model of spondyloarthritis
Source: Arthritis Res Ther. 2019 Feb 4;21:46. doi: 10.1186/s13075-019-1827-9 (PMC6360689; doi:10.1186/s13075-019-1827-9)

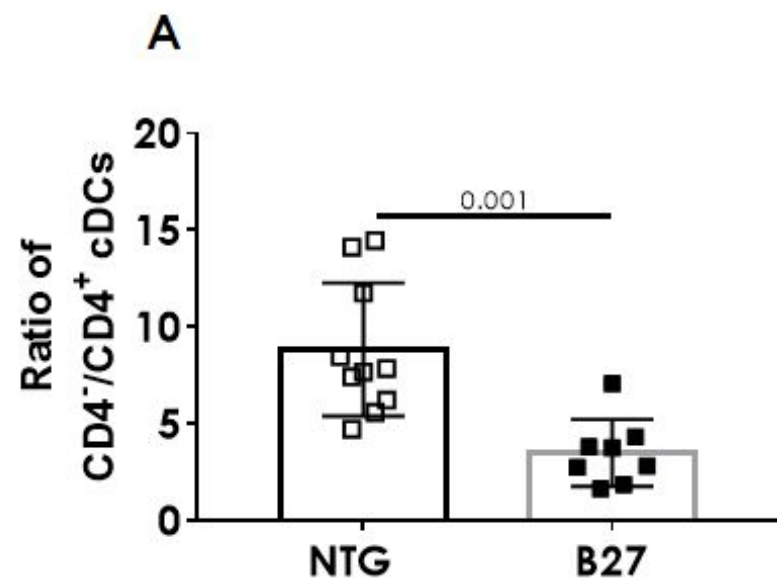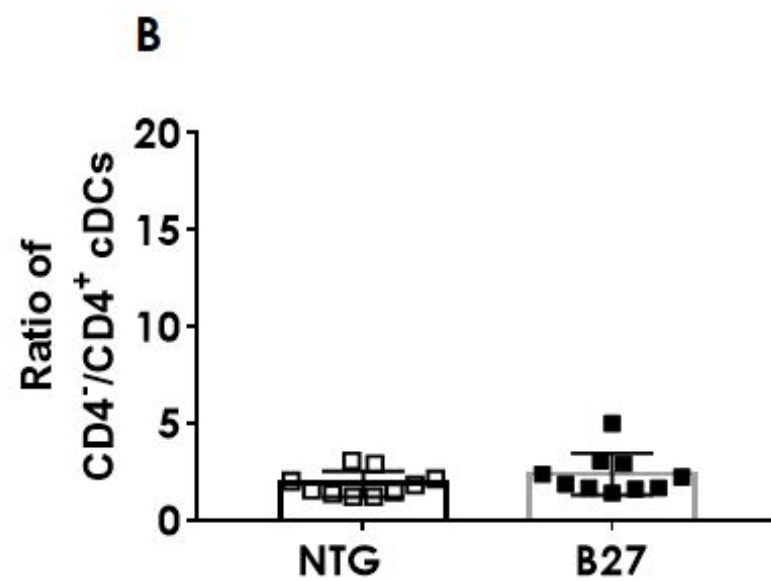

Supplement: Supplementary file 1 — Figure S1. Ratio of CD4-/CD4+ cDCs is reduced in the spleen but not in MLN from B27-Tg rat. Splenic CD103+ cDCs were isolated from NTG and B27-Tg rats with established disease and analyzed by flow cytometry. The ratio of CD103+CD4−/CD103+CD4+ cDCs was analyzed among live cells in the spleen (A) or in MLN (B). This experiment was repeated 10 times. Bars show the mean ± SEM. Data were analyzed by unpaired Student’s t test. (PDF 39 kb) [file 13075_2019_1827_MOESM1_ESM.pdf]

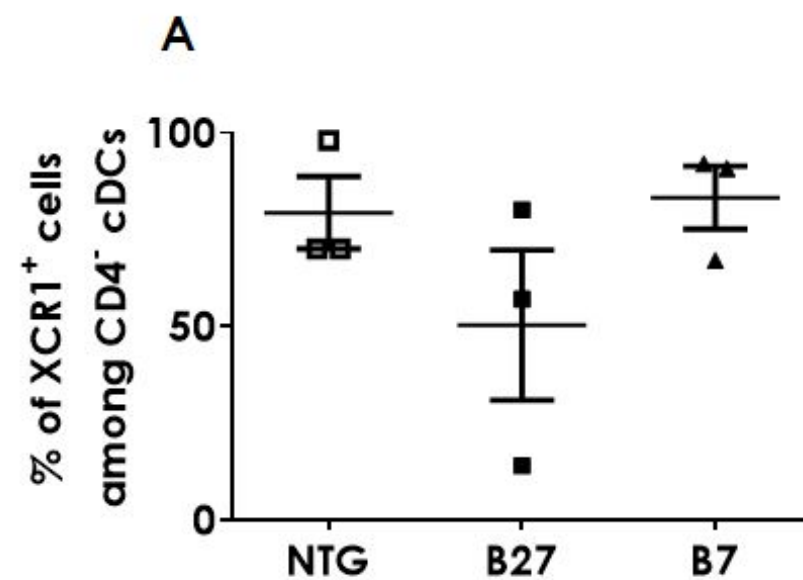

Supplement: Supplementary file 2 — Figure S2. Frequency of splenic XCR1+ cDCs is similar in B7-Tg rats than in NTG rats at 6 month. CD103+cDCs were isolated from the spleen of 6-month-old NTG, B7-Tg, and B27-Tg rats. XCR1 frequency was evaluated in CD4− cDCs by flow cytometry. The graph shows the frequency of XCR1+ cDCs among CD4− CD103+ cDCs in NTG, B7-Tg, and B27-Tg rats. This experiment was repeated 3 times. Bars show the mean ± SEM. (PDF 31 kb) [file 13075_2019_1827_MOESM2_ESM.pdf]

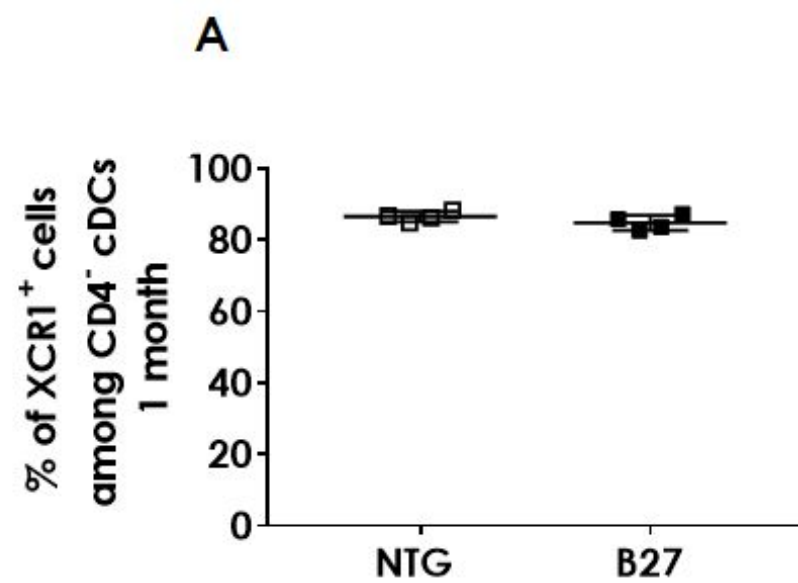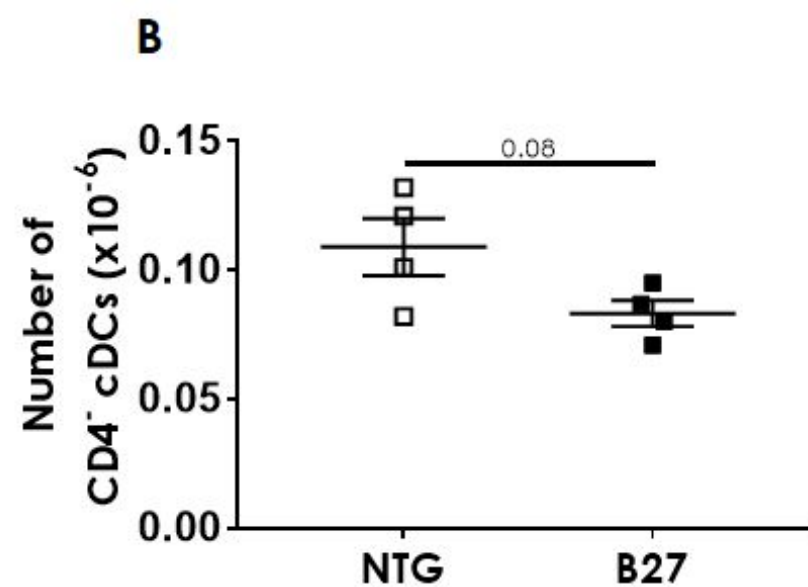

Supplement: Supplementary file 3 — Figure S3. The number of splenic XCR1+ cDCs is marginally decreased in B27-Tg rat at 1 month. CD103+ cDCs were isolated from the spleen of 1-month-old NTG and B27-Tg rats. (A) The graph shows the frequency of XCR1+ cDCs among CD4− CD103+ cDCs in NTG and B27-Tg rats. (B) The graph shows the number of CD4− cDCs in NTG and B27-Tg rats. This experiment was repeated 4 times. Bars show the mean ± SEM. Data were analyzed by unpaired Student’s t test. (PDF 40 kb) [file 13075_2019_1827_MOESM3_ESM.pdf]

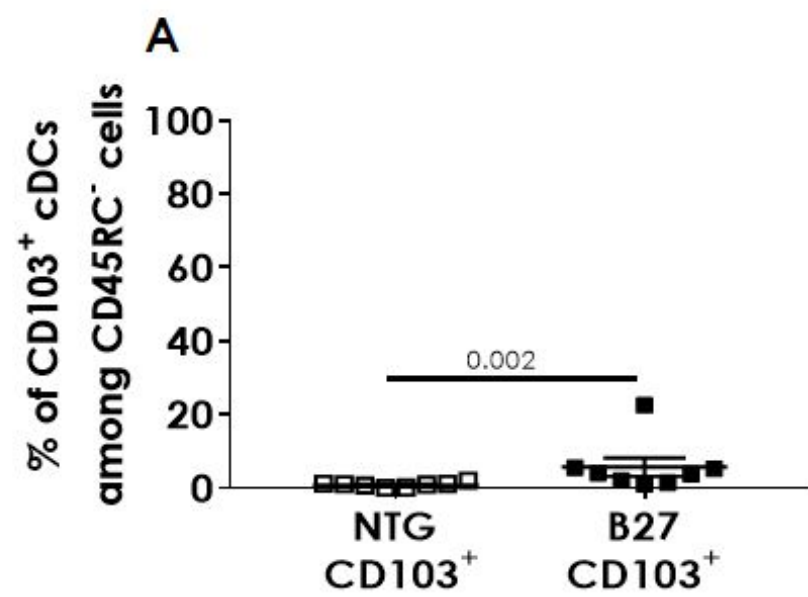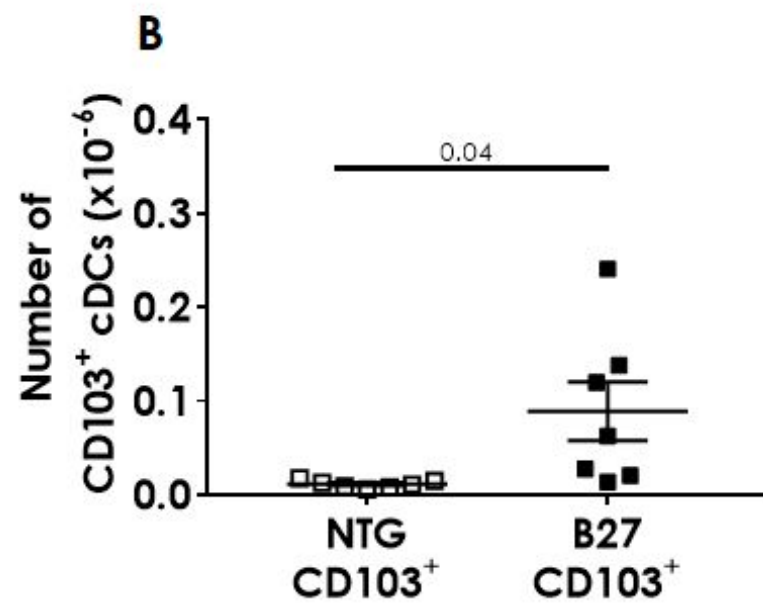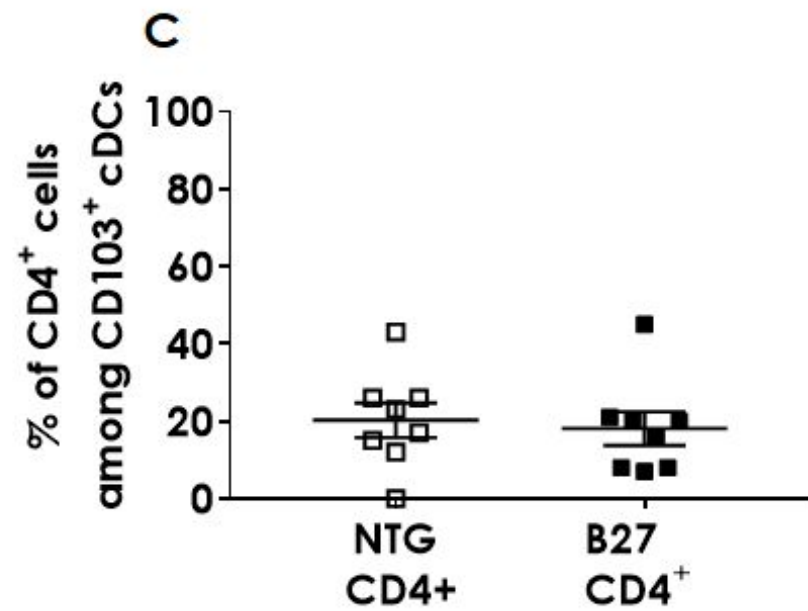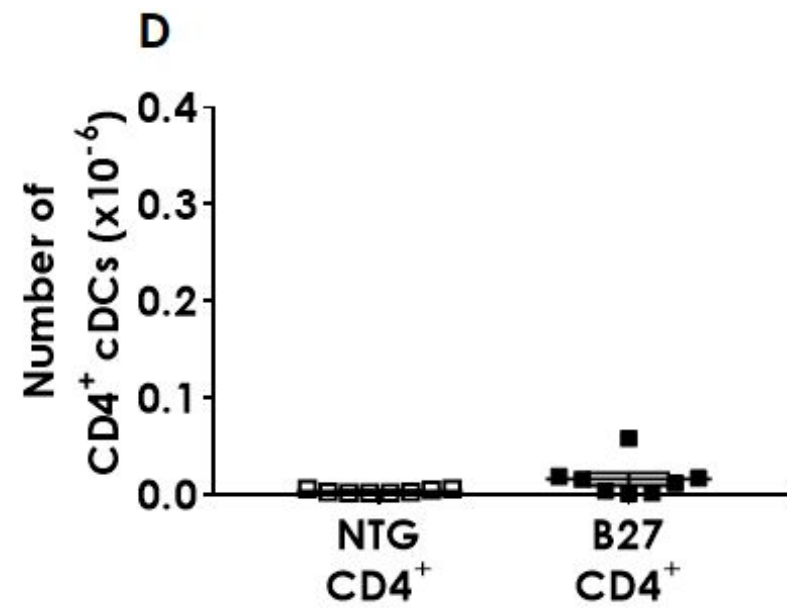

Supplement: Supplementary file 4 — Figure S4. The whole population of CD103+ cDCs is increased but not the CD4+ cDCs, in B27-Tg rat colonic lamina propria. Mononuclear cells were isolated form the colonic lamina propria and stained with anti-CD103 and anti-CD45 mAbs. The graphs show the frequency (A) and absolute number (B) of CD103+ cDCs among CD45RC− cDCs in NTG and B27-Tg rats. The graphs show the frequency (C) and absolute number (D) of CD4+ cDCs among CD103 + cDCs in NTG and B27-Tg rats. This experiment was repeated 7–8 times. Bars show the mean ± SEM. Data were analyzed by unpaired Student’s t test. (PDF 66 kb) [file 13075_2019_1827_MOESM4_ESM.pdf]

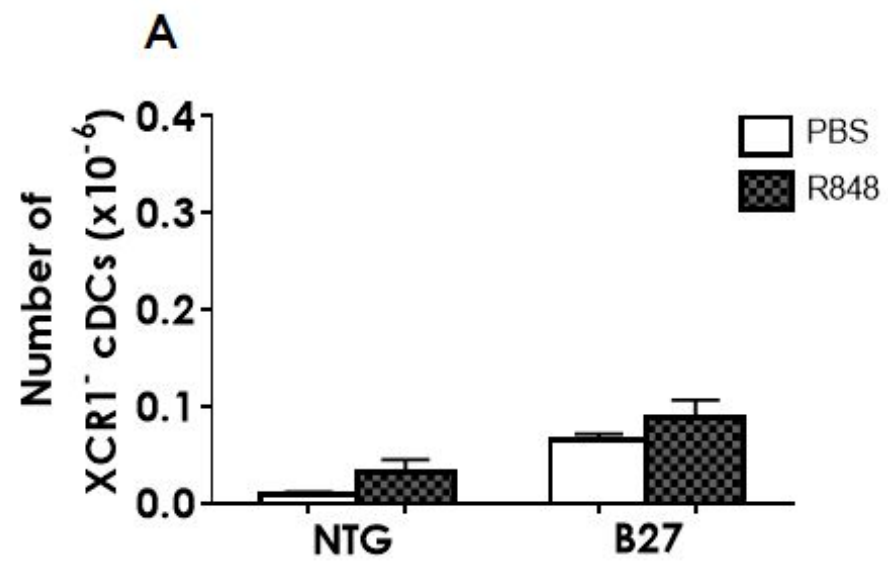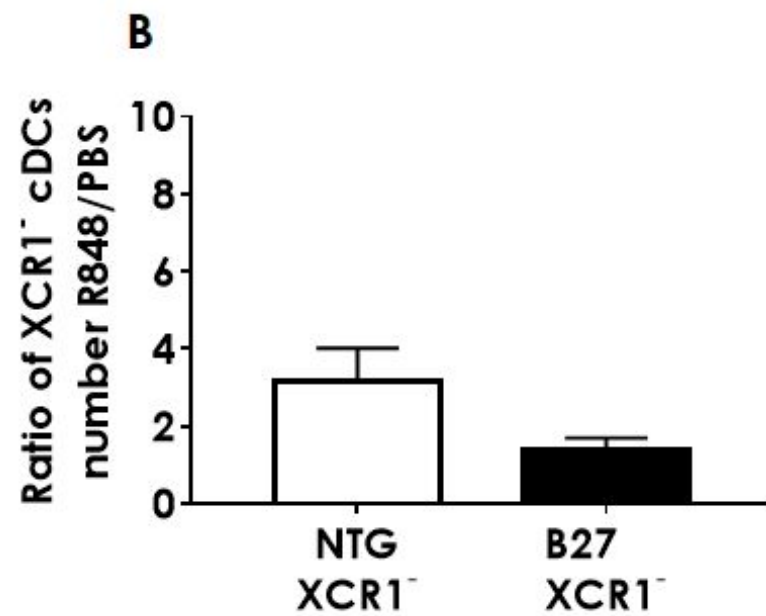

Supplement: Supplementary file 5 — Figure S5. Number of XCR1− cDCs in 6-month-old B27-Tg rat MLN after TLR-7 stimulation. Low-density cells were isolated from 6-month-old NTG and B27-Tg rats MLN that had been fed 5 h before with the TLR-7 agonist R-848 to activate DC migration from the intestine to the afferent lymph. Control NTG and B27-Tg rats received PBS (A) The graph shows the absolute number of XCR1− cDCs among CD4− CD103+ cDCs in R-848- and PBS-fed NTG and B27-Tg rats. (B) The graph shows the ratio of XCR1− cDC numbers between R848- and PBS-fed conditions in NTG and B27-Tg rats. This experiment was repeated 5 times. Bars show the mean ± SEM. Data were analyzed by unpaired Student’s t test. (PDF 42 kb) [file 13075_2019_1827_MOESM5_ESM.pdf]
